# Potential predictors of COVID-19 disease infection and severity in Egypt

**DOI:** 10.1038/s41598-025-34444-y

**Published:** 2026-01-29

**Authors:** Wedad M. Abdelraheem, Raghda Raouf Shady, Wafaa K. M. Mahdi, Mohamed Ibrahim Bassyouni, Heba S. Kamel, Yosra M. Mousa, Shiamaa F. Kamel, Manal Mohamed Saber, Soha S. Abdelrahim

**Affiliations:** 1 Medical Microbiology and Immunology Department, Faculty of Medicine, Minia University, Minia, Egypt; 2 Biochemistry Department, Faculty of Medicine, Minia University, Minia, Egypt; 3 Chest Department, Faculty of Medicine, Minia University, Minia, Egypt; 4 Internal Medicine Department, Faculty of Medicine, Minia University, Minia, Egypt; 5 Clinical Pathology Department, Faculty of Medicine, Minia University, Minia, Egypt

**Keywords:** COVID-19, CD4 + :CD8 + ratio, PD-1, IL-1β, IL-6, miR-146a, miR-133a, Biochemistry, Immunology, Microbiology, Medical research, Molecular medicine

## Abstract

This study aimed to detect the changes in certain immunological parameters and miRNAs in COVID-19 cases with various degrees of disease severity and compare these changes in cases to healthy controls. This study was conducted on 45 COVID‐19 patients and 45 healthy controls. The flow cytometry was conducted to study the number of CD4 + and CD8 + T cells and evaluate the level of the PD-1 marker on their surfaces for all study participants. The determination of IL-1β and IL-6 in serum for all study subjects was done by ELISA test. Relative gene expression quantitation of miR-146a and miR-133a was performed by reverse transcriptase real-time PCR (RT-PCR). The numbers of CD4 + and CD8 + T cells were dramatically reduced in COVID-19 patients, especially in severe to critical patients, with an increase in the CD4 + :CD8 + ratio. T cells from COVID-19 patients had significantly higher levels of the exhausted marker PD-1. Measurement of IL-1β and IL-6 serum levels among cases group showed a highly significant increase in their mean concentration levels in comparison with the control group. Studying the difference in serum levels of IL-1β and IL-6 among different degrees of disease severity showed a significant decrease in their mean concentration levels among the mild to moderate group in comparison with the severe to critical group. The results also showed a significant decrease in miR-146a and a significant increase in miR-133a expression in COVID-19 patients compared to healthy controls. Reduced T cell counts, increased CD4 + :CD8 + ratio, higher levels of the PD-1 marker, elevated serum levels of the pro-inflammatory cytokines, and decreased miR-146a and increased miR-133a gene expressions could be used as potential markers in the assessment of COVID-19 infection and severity.

**Supplementary Information:**

The online version contains supplementary material available at 10.1038/s41598-025-34444-y.

## Introduction

Severe acute respiratory syndrome-related coronavirus (SARS-CoV-2) is a novel, highly infectious human beta coronavirus. It was first detected in late 2019 and precipitated an ongoing worldwide pandemic^1^. COVID-19 is a respiratory disease transmitted either through exposure to micro-droplets from infected individuals or by contact through contaminated fomites affecting the lung parenchyma^2^. Most COVID-19 patients experience mild to moderate symptoms such as fever, cough, muscle pain, fatigue, and minor symptoms such as skin rash, loss of taste, headache, and gastrointestinal complications. Furthermore, blood and biochemistry tests often show abnormalities, indicating that SARS-CoV-2 infection affects various systems, including the cardiovascular, gastrointestinal, hematopoietic, and immune systems. It is estimated that about 15% of patients may develop severe pneumonia, and approximately 5% may progress to acute respiratory distress syndrome, septic shock, or multiple organ failure^3^. Research on the immune response to COVID-19 infection and vaccination is still incomplete. It is unclear if the viral infection, immunological host response, or both contribute to the disease severity. Understanding these changes can provide valuable insights into the prognosis and potential complications of the disease and help with developing prevention and treatment strategies^4^.

Examination of the CD4/CD8 ratio as a quantitative trait can be important to patient care, as it might be used as a prognostic risk factor for COVID-19 infection. T-cell exhaustion is recognized as a unique state of CD8 + T cell differentiation, marked by traits that make it a central cause of immune dysfunction in chronic viral infections and cancer. This state is characterized by the persistent expression of inhibitory receptors like programmed cell death-1 (PD-1), which, when highly expressed, can disrupt signaling pathways and suppress lymphocyte proliferation and differentiation^5^.

Studying the proinflammatory cytokines like IL-1, IL-6, TNF-α, and IFN-γ in COVID-19 patients is important as the abnormal elevation of these cytokines can lead in certain cases to a cytokine storm^6^. Therefore, identifying these biomarkers in early stages of the disease has important implications for treatment to decrease mortality for COVID-19^7^. A group of miRNAs can regulate inflammation (referred to as inflamma-miRs) such as miR-146a, miR-21-5p, and miR-126-3p. The miR-146a gene is activated in human monocytes when inflammation occurs. It plays a vital role in regulating viral replication, while being a crucial component of the inflammatory response and the T-lymphocyte-mediated adaptive immune response^8^. Also, distinct circulating miRNAs, including miR-1, miR-21, miR-133a, miR-208b, and miR-499a, show increased levels in acute myocardial infarction. These cardio-metabolic microRNAs’ expression patterns were associated with clinical severity and mortality in COVID-19 patients^9^. As the COVID-19 pandemic has become a global health crisis, predicting the severity of the disease early on is crucial for reducing mortality rates and curbing its spread. In this study, we aimed to detect the number of CD4 + and CD8 + T cells, evaluate the exhaustion levels of these T-cells by detecting the PD-1 marker on their surfaces in COVID-19 patients with various degrees of disease severity, and compare these changes with healthy controls. Also, we aimed to compare the expression level of two miRNAs, miR-146a and miR-133a, and the serum level of two proinflammatory cytokines, IL-1β and IL-6, in COVID-19 patients with various degrees of disease severity.

## Materials and methods

### Study participants

The study was conducted at the Microbiology and Immunology Department in cooperation with the Clinical Pathology Department of the Faculty of Medicine, Minia University, Egypt. Study participants were enrolled through the Chest Diseases Department at Minia University Hospital. This case–control observational study was carried out during the period from February 2022 to November 2023 on 45 COVID‐19 patients and 45 healthy controls. The control group consisted of healthy individuals with no evidence of viral infections, immune-related disorders, or chronic illnesses. They were matched to the cases regarding age, sex, and residential area.

### Inclusion criteria

All cases were confirmed positive for SARS-CoV-2 using the genesig real-time PCR detection kit for SARS-CoV-2 applied on RNA extracted from upper respiratory tract samples retrieved from nose or throat swabs.

COVID-19 patients were classified into mild-moderate cases group (N = 25) and severe-critical cases group (N = 20) based on the medical records of the patients and according to the Management Protocol for COVID-19 patients released by the Egyptian Ministry of Health^10^, as follows:

1. *Mild cases*: Symptomatic cases (fever and/or respiratory symptoms) with lymphopenia or leucopenia with no radiological signs of pneumonia.
2. *Moderate cases*: The patient has radiological signs of pneumonia manifestations associated with leucopenia or lymphopenia.
3. *Severe cases*: Respiratory Rate (RR) > 30 cycles, Saturation of Oxygen (SaO2) < 92% at room air, Partial Arterial pressure of Oxygen to Fraction of inspired Oxygen (PaO2 /FiO2) ratio < 300 kPa, Chest radiology showing more than 50% lesion or progressive lesion within 24 to 48 h.
4. *Critical cases*: RR > 30 cycles, or SaO2 < 92%, or PaO2/FiO2 ratio < 200 kPa despite Oxygen Therapy.

### Exclusion criteria

Any medical state that may cause changes in the immune markers and serum levels of proinflammatory cytokines was excluded from the study. These excluded medical conditions include any other viral infection, immune disorders, and chronic diseases.

A medical history was taken from all cases, considering age, sex, residence, smoking, history of any co-existing diseases affecting the immune response, and history of vaccination against SARS-CoV-2. The patients were classified according to the vaccination status into four groups: (1) The unvaccinated group included individuals who did not receive any COVID-19 vaccines before becoming infected or received COVID-19 vaccines on the day of infection. (2) The partially vaccinated group included individuals who had not completed full primary vaccination or completed primary vaccination less than 14 days before becoming infected. (3) The full vaccination group included individuals who completed 2 doses of inactivated vaccine, 1 dose of adenovirus-vectored vaccine, or 3 doses of recombinant-subunit vaccine 14 or more days before becoming infected. (4) The booster dose group included individuals who received a booster dose 7 or more days before becoming infected^11^.

### Blood sampling protocol

Blood samples were collected from all individuals, with five milliliters divided into two tubes. Two milliliters were collected in EDTA vacutainer collection tubes and processed within four hours of blood withdrawal for flow cytometry studies. The remaining three milliliters of blood were collected in clot activator vacutainer tubes and centrifuged at 1,800 rpm for five minutes to separate the blood serum. The supernatant serum was stored at − 80 °C to be used to detect inflammatory cytokines by the ELISA technique and the determination of serum miRNAs levels by PCR.

### Flow cytometry technique

#### Antibodies

 The identification of PD-1^+^ CD4^+^ T cells% and PD-1^+^ CD8^+^ T cells% was assessed using commercially synthesized monoclonal antibodies. A CD4 monoclonal antibody (400 μg/mL, catalog no. 300506, BioLegend, San Diego, CA, USA); CD3 monoclonal antibody (200 μg/mL, catalog no. 300406, clone UCHT1, BioLegend); CD8 monoclonal antibody (100 μg/mL, catalog no. 344704, clone SK1, BioLegend); PD-1 monoclonal antibody (400 μg/mL, catalog no. 329906, clone EH12.2H7, BioLegend). The baseline for the PD-1 staining was determined using an isotype-matched control (PE mouse IgG2a (k) isotype control, catalog no. 402203, BioLegend).

#### Flow cytometry analysis

 For each sample, 3 tubes were labeled, one tube for fluorescein isothiocyanate (FITC)-conjugated anti-CD3^+^, another tube for fluorescein isothiocyanate (FITC)-conjugated anti-CD4^+^, allophycocyanin (APC)-conjugated anti-CD8^+^, and phycoerythrin (PE)-conjugated anti-PD-1^+^. The last tube was for fluorescein isothiocyanate (FITC)-conjugated anti-CD4^+^, allophycocyanin (APC)-conjugated anti-CD8^+^, and phycoerythrin PE mouse IgG2a (k) isotype control. All antibodies were purchased from BioLegend (San Diego, CA, USA). Briefly, five microliters of antibodies were added to 100 µL of peripheral blood and incubated for 30 min in the dark at room temperature. Then, one mL of red cell lysis buffer was added, vortexed, and incubated for 10 min in the dark at room temperature. Sample centrifugation was performed for about five minutes at 1200 rpm, and the supernatant was removed. Subsequently, 1 mL of washing phosphate-buffered saline (PBS) solution was added to every tube, mixed, centrifuged at 1200 rpm for five minutes, and the supernatant was removed. Then, 300 µl of PBS were added to the cells to resuspend them for flow cytometry analysis. BD-FACS FLOW (Argon laser, BD Biosciences, San Jose, CA, USA) was used for cell analysis using the Cell Quest Program. Cell surface expression of CD3^+^ was determined at 525 nm-wavelength laser excitation, and emitted fluorescence was monitored with a detector optimized to collect peak emissions at 504-541 nm. CD8 + , CD4 + , and PD-1 + cell surface expressions were determined at 661, 525, and 578 nm wavelengths, respectively. The lymphocyte gating was assessed by the forward vs. side scatter (FSC/SSC) plot. CD4^+^%, CD8^+^%, PD-1^+^ CD4^+^%, and PD-1^+^ CD8^+^% cells were assessed^12^. Manual gating was done on the flow cytometry data. The lymphocyte populations were identified and gated from FSC and SSC dot plots (Forward scatter vs. side scatter technique). We have identified the T-cell population from the lymphocyte population based on CD3 expression. T-helper and cytotoxic T-cell populations were identified based on CD4 and CD8 expression, respectively, in the CD3-positive T-cell population. To identify exhausted T-cells, we further looked at the expression of PD-1 on the surface of CD4^+^ and CD8^+^ T-cells. The cutoff values were calculated using the isotypic controls as a guide. Unstained cells were employed as a negative control for every patient^13^.

### Determination of IL-1β and IL-6 in serum by ELISA procedure

Determination of IL-1β and IL-6 in serum was done by Huma reader 3700, Germany, using the following ELISA kits: Human IL1b, Cat: ELK1270, and Human IL6, Cat: ELK1156 (ELK Biotechnology, USA). The procedure was done according to the kit protocol, and the color change was measured spectrophotometrically at 450 nm (Huma reader 3700, Germany). All experiments were performed in triplicate, and the mean was calculated to ensure accuracy. Supplementary Fig. S1 shows the standard curve used to calculate serum IL-1β and IL-6, respectively.

### Relative quantitation of miR-146a and miR-133a genes by real-time PCR

Relative quantitative detection of miR-146a and miR-133a genes was performed by reverse transcriptase real-time PCR (RT-PCR) in a Real-time PCR system (Applied Biosystem 7500 Fast, California, USA). Normalizing the amount of target miRNA by using a suitable endogenous reference gene is necessary. The miR-16 gene was chosen to be an endogenous reference gene as it was found to be expressed continuously at a constant level in human cells^14^.

#### RNA extraction

 Purification of total RNA, including microRNAs, from serum was done using GENEzol™ TriRNA Pure Kit. The quantity of the extracted RNA was assessed by measuring the absorbance with a spectrophotometer (Genova, USA). The ratio of the absorbance at 260 nm and 280 nm was used to determine the purity of the extracted RNA; the result within the 1.8 to 2 range was considered acceptable purity.

#### RT-PCR

 RT-PCR was done on the extracted RNA using TOP real™ One-step RT qPCR Kit (SYBR Green with low ROX), and forward and reverse primers for the reference and the target genes. The sequence of primers used in this study is listed in Supplementary Table S1^15^. The reaction mixture was prepared, and PCR conditions were followed according to the kit protocol. All experiments were performed in triplicate, and the mean was calculated to ensure accuracy. A melting curve was performed to increase the specificity of the PCR product. Once PCR reactions are completed, expression analysis is calculated by the relative quantification (RQ) method. The expression of miRNAs was reported as the ΔCt value, calculated by the following formulas^16^: ∆Ct = Ct (gene of interest)—Ct (housekeeping gene). ∆∆Ct = ∆Ct (Sample)—∆Ct (Control average). Fold gene expression (RQ) = 2^-∆∆Ct.

### Statistical analysis

The data analysis was conducted using IBM SPSS statistical package software version 28 (IBM Corp., computer software, Armonk, NY, USA). Descriptive statistics were used to compute the interquartile range (IQR), median, and frequency for quantitative non-parametric data, while percentages and numbers were employed for qualitative data. The Mann–Whitney test was used to compare quantitative non-parametric data from two different groups. For qualitative data, Fisher’s exact or chi-squared analysis was performed to compare the two groups. Pearson’s correlation was determined between the continuous variables. Receiver Operating Characteristic (ROC) curves were utilized to identify the optimal cutoff value and to evaluate the specificity, sensitivity, negative predictive value (NPV), positive predictive value (PPV), and overall accuracy of the various variables. The area under the curve (AUC) was utilized to investigate the prediction accuracy of NHL markers. The p-value cutoff was less than 0.05.

## Results

### Demographic data

This study was performed on 45 adult COVID-19 patients and 45 adult healthy control subjects. The mean age of the two groups was 49.2 ± 16.869 and 48.3 ± 15.2 years, respectively. Among the 45 COVID-19 cases, 25 (55.6%) were classified as mild to moderate, whereas the remaining 20 (44.4%) were classified as severe to critical. The study revealed no significant difference in disease severity between male and female patients. However, it did vary significantly based on age (P-value < 0.001). Young adults accounted for 56% of those with mild to moderate symptoms, while elderly individuals accounted for 75% of the severe to critical cases. (Supplementary Table S2). Detecting smokers from non-smokers in the studied cases, the study revealed that 26.7% (n = 12) of the cases were smokers. Out of these, 3 cases belonged to the mild to moderate group, and the remaining 9 smokers were part of the severe to critical group with a significant P-value < 0.001 (Supplementary Table S2). According to the study, there is a significant difference in the disease severity between rural and urban areas, with a P-value of less than 0.001(Supplementary Table S2).

Studying the vaccination status of COVID-19 patients revealed a significant difference in the vaccination status among different degrees of disease severity (Supplementary Table S2). All the severe to critical patients were partially vaccinated (100%). However, 14 out of 25 cases (56%) in the mild to moderate group were fully vaccinated, and 11 cases (44%) had their booster dose vaccination (Supplementary Table S2).

### Clinical and radiological data

Clinical symptoms such as fever and/or cough were observed in 76% of patients with mild to moderate disease and in 100% of those with severe to critical illness, indicating a statistically significant difference (P-value < 0.05) among different levels of disease severity. Studying the respiratory signs among COVID-19 cases, with different degrees of disease severity, showed that 36% had an increase in RR with normal SO2. No cases (0%) in the mild to moderate group had problems with the O2 saturation. All the severe to critical group had abnormalities with the O2 saturation, where 50% of cases showed an increase in RR with a decrease in SaO2 at room air, and 50% also showed a decrease in PaO2/FiO2 ratio despite oxygen therapy.

Radiological findings on CT scans in COVID-19 patients varied significantly depending on the severity of the disease. In the mild to moderate group, 64% had normal CT results, 36% exhibited signs of pneumonia, and none (0%) showed ground-glass opacities. The severe to critical group was divided according to radiological findings: 50% of cases had radiological signs of pneumonia, and the other 50% had ground glass opacities with interstitial fibrosis. In contrast, no severe to critical cases (0%) showed normal CT findings with no radiological signs of pneumonia.

### Complete blood count data

The blood test data of the 45 COVID-19 patients collected from the medical records of the patients in the hospital are presented in the supplementary Table S3. All the cases studied showed a red blood cell (RBC) count and Hemoglobin (HB) level within the normal range. Thrombocytopenia was found only in 40% of the severe to critical group, while all the rest of the studied cases showed a normal platelet count. Leukocytosis was present in all cases of the severe to critical group (100%), while it was found in only 8 percent of the mild to moderate group. Lymphopenia was present in 89% of all the patients studied, accounting for 92% of the mild to moderate group and 85% of the severe to critical group, with no significant difference among different degrees of disease severity. Neutrophilia was present in all individuals in the severe to critical group and 36% in the mild to moderate group. Monocytopenia was found only in 52% of the mild to moderate group and was absent in the remaining cases studied. There was a tendency for neutrophils to increase and lymphocytes to decrease with the aggravation of the disease. The median neutrophil-to-lymphocyte ratio (NLR) in COVID-19 patients was 7.8 ± 2.4 in the mild to moderate group and increased to 19.2 ± 8 in the severe to critical group.

The accuracy of the association of the presence of leukocytosis, neutrophilia, lymphopenia, elevated NLR and thrombocytopenia in severe to critical cases compared to the mild to moderate group done by Receiver Operating Characteristic (ROC) curve analysis showed that leukocytosis, neutrophilia, elevated NLR and thrombocytopenia can be used as a diagnostic biomarker for the prediction of disease severity with excellent discriminating efficiency (Area Under the Curve (AUC) = 0.9–1 and P-value < 0.001), while lymphopenia failed to discriminate between degrees of disease severity (AUC = 0.591 and P value = 0.299) as shown in Table 1.

**Table 1:** The accuracy of the association of CBC data with COVID-19 disease severity.

	AUC	95% confidence interval		Sensitivity (%)	Specificity (%)	Cut off	p-value
		Lower bound	Upper bound				
Leukocytosis	1	1	1	100	100	12,750	< 0.001*
Neutrophilia	1	1	1	100	100	10,550	< 0.001*
Lymphopenia	0.591	0.412	0.770	70	60	937.5	0.299
Elevated NLR	0.983	0.956	1	90	96	11.85	< 0.001*
Thrombocytopenia	0.909	0.829	0.989	100	64	205	< 0.001*

AUC (0.9–1) = excellent.

AUC (0.5–0.6) = fail.

-*Significant level at P value < 0.05.

Significant values are in [bold].

### C-reactive protein (CRP) and D-dimer levels

CRP and D-dimer results were collected from the medical records of the patients in the hospital. Regarding D-dimer level, there was a significant difference in its level among different degrees of disease severity. It was elevated above the normal level in all severe to critical cases included in this study (N = 20, 100%), ranging from 0.8 to 32 mg/dl. In the mild to moderate group, there were 17 cases out of 25 cases (68%) that showed normal D-dimer serum levels, and 8 cases (32%) showed elevation in the D-dimer serum level, ranging from 0.6 to 1.2 mg/dl, as shown in the supplementary Table S4.

Regarding CRP level, there was no difference between degrees of disease severity in its serum level as it was elevated above the normal level in all cases included in this study (100%), ranging from (2.6–22.3 mg/dl) in mild to moderate group with mean and SD (11 ± 6.7), while the range of CRP serum level in severe to critical group was (27.7–76.8 mg/dl) with mean and SD (49.6 ± 13.6) as shown in supplementary Table S4.

**Fig. 1:**
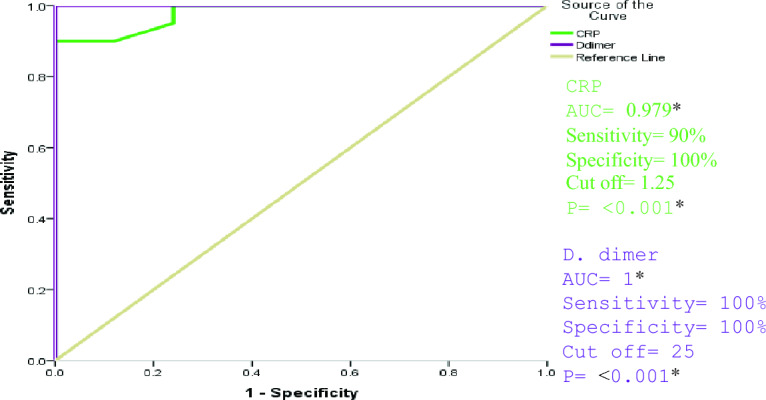
ROC curve analysis for the accuracy of the association between elevated serum levels of CRP and D-dimer with COVID-19 disease severity. AUC, Area under the curve. *AUC (0.9–1) = excellent discriminating efficiency, **AUC (0.7–0.8) = fair discriminating efficiency. *Significant level at P value < 0.05.

The accuracy of the association of elevated serum levels of CRP and D-dimer in severe to critical cases compared to the mild to moderate group was assessed by ROC curve analysis. It showed that elevated serum levels of CRP and D-dimer can be used as diagnostic biomarkers to predict disease severity with excellent discriminating efficiency (AUC = 0.979 and 1, respectively, with P-value < 0.001 for both relations) as shown in Fig. 1.

### Flow cytometric data analysis

**Fig. 2:**
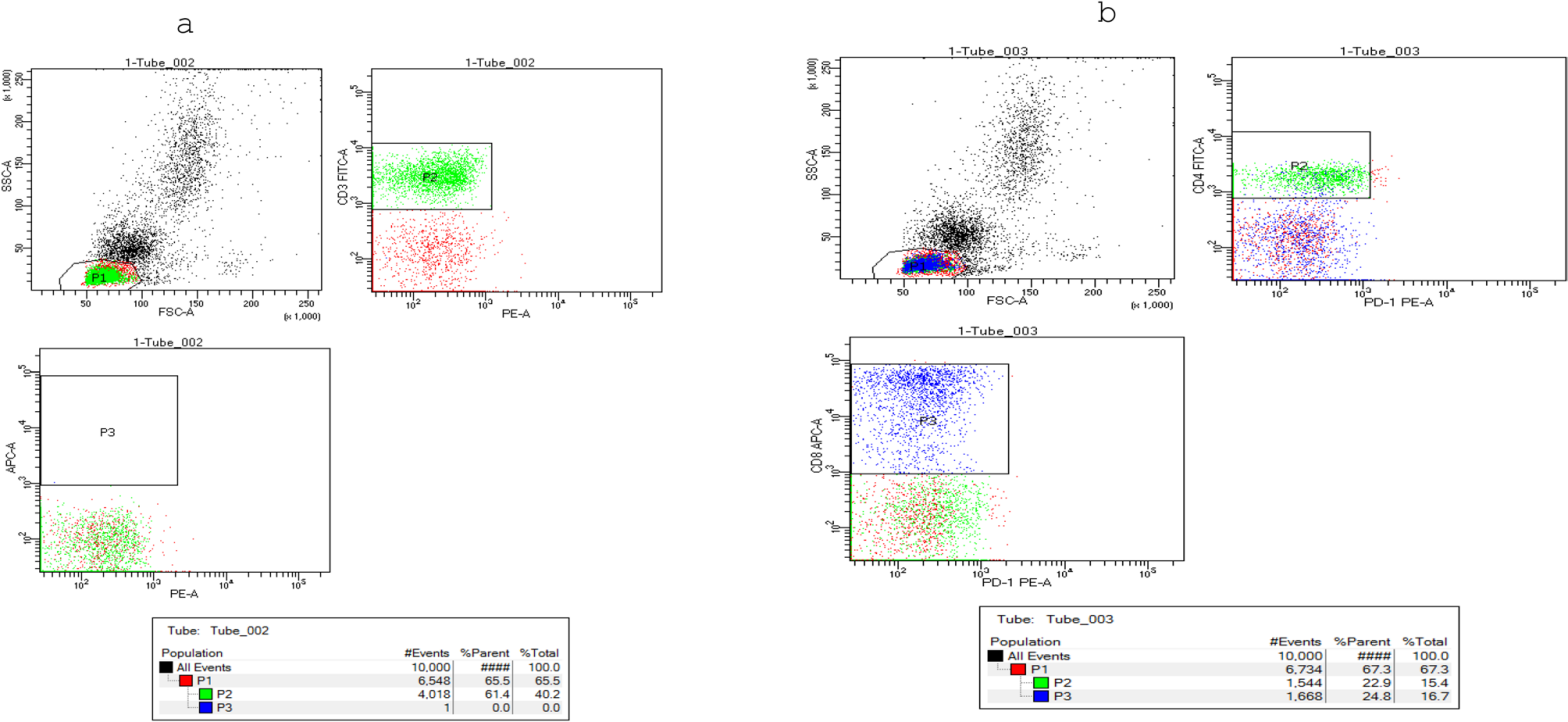
Flow cytometric analysis of expression of PD-1 on CD3 + CD4 + T cells and CD3 + CD8 + T cells. (**a**): Dots-plots of CD3 + cells %, (**b**): Dots-plots of CD4 + PD-1 + cells % and CD8 + PD-1 + cells %.

**Fig. 3:**
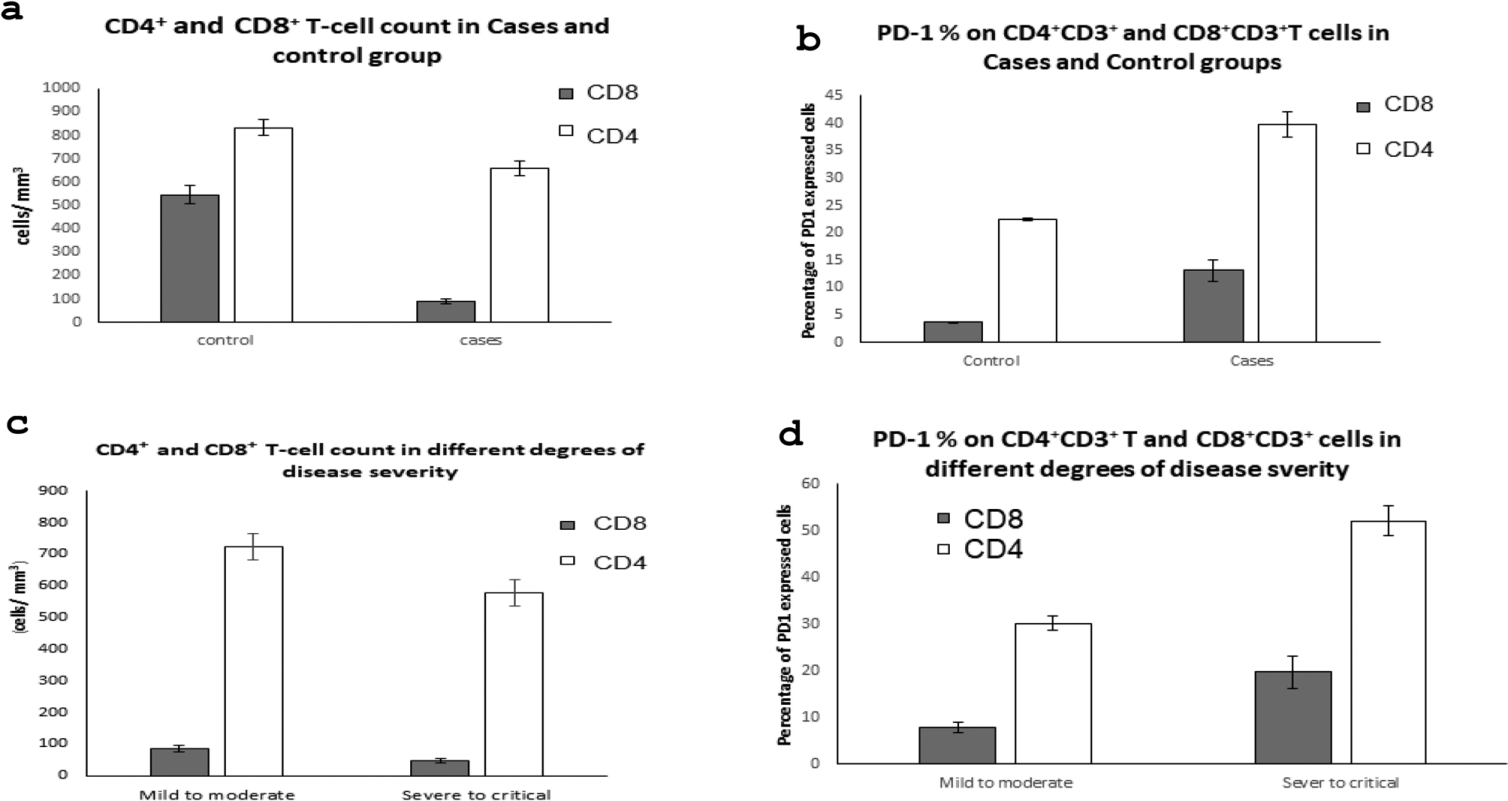
Results of the flowcytometry test.** (a**): CD4 + and CD8 + T-cell count in Cases and control group, (**b**): PD-1% on CD4 + CD3 + and CD8 + CD3 + T cells in Cases and Control group, (**c**): CD4 + and CD8 + T-cell count in different degrees of disease severity, (**d**): PD-1% on CD4 + CD3 + and CD8 + CD3 + T cells in different degrees of disease severity.

All flow cytometric data are shown in Table 2. The analysis of results showed that patients had remarkably low CD4 + and CD8 + T cell counts (Mean = 659 and 69 cells/mm3, respectively) compared to the control group (Mean = 833 and 34 cells/mm3, respectively). The mean value of CD4 + and CD8 + T-cell counts decreased to 577 and 48 cells/mm3, respectively, in the severe to critical group compared to the mild to moderate group (Mean = 725 and 86 cells/ mm3, respectively). The CD4:CD8 ratio was significantly elevated in cases compared to the healthy control group (Mean = 13 and 2.5, respectively). At the same time, it was higher in the mild to moderate group than in the severe to critical group (Mean = 17 and 10, respectively), demonstrating a lack of CD4 + and CD8 + T cell expansion, which were significantly associated with disease severity. Flow cytometry analysis illustrated in Fig. 2 showed markedly higher percentages of PD-1 + CD8 + and CD4 + T cells in COVID-19 patients, especially the severe to critical group, compared to the control group as shown in Fig. 3. This indicates that SARS-CoV-2 can drive T cell exhaustion in COVID-19 patients, particularly among those with severe to critical degrees of disease.

**Table 2:** Flow cytometric data in COVID-19 patients and control group.

	The detected cells	Control group N = 45	Cases group N = 45	P value	R	Mild to moderate cases N = 25	Severe to critical cases N = 20	P value	R
CD4^+^ T-cells Count (cells/ mm^3^**)**	Range	500–1200	255–1228	< 0.001	− 0.8*	255–1228	284–882	0.09	− 0.3
	Mean ± SEM	833.3 ± 33.7	658.8 ± 31.7			724.8 ± 50.3	577.3 ± 34.1		
PD-1% on CD3^+^**CD4**^+^ T cells	Range	21–24	21–80	< 0.001	0.56**	21–49	36–80	< 0.001	0.69
	Mean ± SEM	22.5 ± 0.16	39.8 ± 2.3			30.1 ± 1.5	52.1 ± 3.2		
CD8^+^ T-cells Count (cells/ mm^3^**)**	Range	200–1000	16 -165	< 0.001	− 0.8*	16–165	16–109	< 0.001	− 0.4
	Mean ± SEM	546.7 ± 38.9	68.9 ± 7			85.6 ± 10.5	48.1 ± 6		
PD-1% on CD3^+^**CD8**^+^ T cells	Range	3–5	3–69	< 0.001	0.47**	3–11	3–69	0.002	0.5
	Mean ± SEM	3.7 ± 0.1	13.1 ± 1.8			7.8 ± 1.1	19.7 ± 3.4		
CD4:CD8 ratio	Range	1–4	2–48	< 0.001	0.43**	7–48	5–22	0.002	− 0.38
	Mean ± SEM	2.5 ± 0.2	13 ± 1.6			17 ± 2.4	10 ± 1.2		

*r (0.7–1) = strong correlation.

**r (0.3–0.7) = moderate correlation.

***r (< 0.3) = weak correlation.

-Significant level at P value < 0.05.

**Fig. 4:**
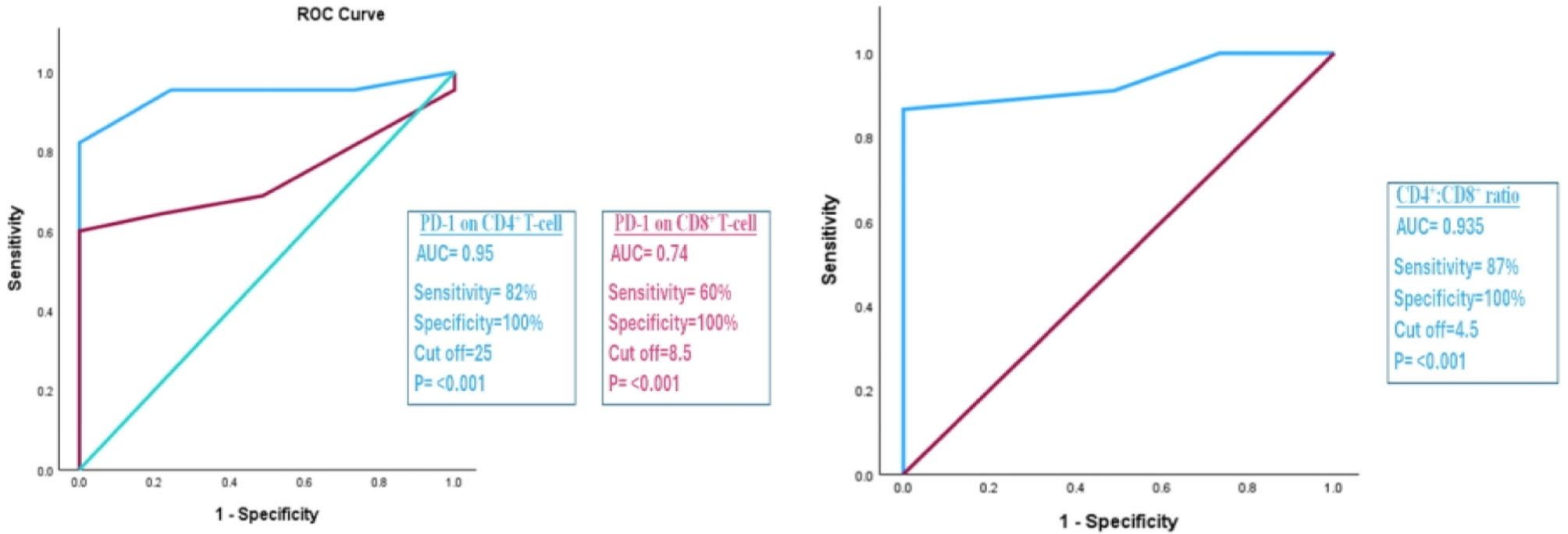
Roc curve analysis for the accuracy of the association between elevated CD4:CD8 ratio and T-cell exhaustion with COVID-19 infection. AUC, Area under the curve. *AUC (0.9–1) = excellent discriminating efficiency, **AUC (0.7–0.8) = fair discriminating efficiency. *Significant level at P value < 0.05.

The accuracy of the association between the increased CD4^+^:CD8^+^ ratio and the increased expression of PD-1 on CD4^+^ T-cells with COVID-19 infection was found to be a diagnostic marker with excellent discriminating efficiency between cases and control groups (AUC = 0.935 and 0.945, respectively)**.** However, increased expression of PD-1 on CD8^+^ T-cells showed a fair discriminating efficiency (AUC = 0.74) as shown in Fig. 4**.**

### The serum levels of IL-1β and IL-6 cytokines

**Fig. 5:**
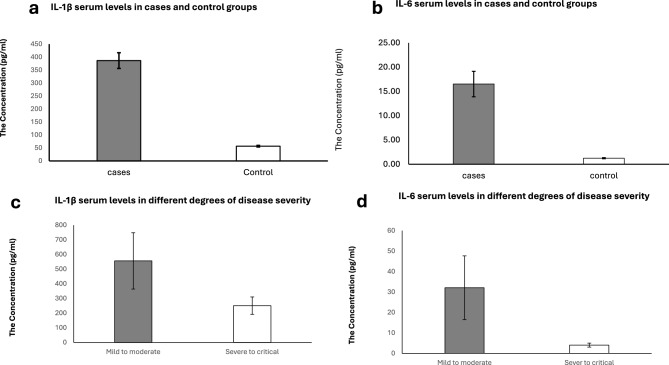
The level of IL-1β and IL-6 among the study participants: (**a**) The mean serum level of IL-1β in cases and control groups, (**b**): The mean serum level of IL-6 in cases and control groups, (**c**): IL-1β serum levels in different degrees of disease severity, (**d**): IL-6 serum levels in different degrees of disease severity.

The serum IL-1β and IL-6 concentration levels were measured in both the COVID-19 case group and the control group using an ELISA kit. Serum IL-1β levels in the COVID-19 patient group ranged from 131.05 to 943.9 pg/ml, showing a highly significant elevation in mean concentration compared to the control group, which ranged from 26.3 to 81.05 pg/ml, with a P-value of less than 0.001. Similarly, serum IL-6 levels in the COVID-19 patient group ranged from 0.18 to 64 pg/ml, showing a significant elevation compared to the control group as shown in Fig. 5, which ranged from 0.16 to 2.5 pg/ml, with a P-value of less than 0.001. Mean concentrations ± SEM of serum IL-1β and IL-6 cytokine levels in the COVID-19 case group and control group are shown in Table 3.

**Table 3:** The mean value ± SEM of the concentration levels of serum cytokines in COVID-19 cases groups and control group:

	Data	Control group N = 45	COVID-19 group N = 45	P value	R	Mild to moderate group N = 25	Sever to critical group N = 20	P value	R
IL-1β (Pg/ml)	Range	26.3–81.05	131.05−943.9	< 0.001	0.75*	131.05- 338.7	344.9–943.9	< 0.001	0.76*
	Mean ± SEM	56.7 ± 3.02	386.6 ± 30.3			251.03 ± 59.18	556.1 ± 192.18		
IL-6 (Pg/ml)	Range	0.16–2.5	0.18–64	< 0.001	0.79*	0.18 -14.85	15.06–64	< 0.001	0.55**
	Mean ± SEM	1.25 ± 0.1	16.5 ± 2.6			4.05 ± 0.98	32.1 ± 15.59		

*r (0.7–1) = strong correlation.

**r (0.3–0.7) = moderate correlation.

- Significant level at P value < 0.05.

Analysis of serum IL-1β level among varying degrees of COVID-19 severity revealed that IL-1β levels in the mild to moderate group ranged from 131.05 to 338.7 pg/ml, showing a significantly lower mean concentration compared to the severe to critical group, which ranged from 344.9 to 943.9 pg/ml. This difference was statistically significant, with a P-value of < 0.001, and demonstrated a strong positive correlation (r = 0.76). IL-6 levels were significantly lower in mild to moderate cases (0.18–14.85 pg/ml) compared to severe to critical cases (15.06–64 pg/ml) with P < 0.001as shown in Fig. 5. A moderate positive correlation was observed between IL-6 levels and disease severity (r = 0.55). Mean concentrations ± SEM of serum IL-1β and IL-6 cytokine levels in the mild to moderate group and severe to critical group are shown in Table 3. Comparing IL-1β and IL-6 serum levels among different vaccination statuses of the studied COVID-19 patients showed a significant difference with a p-value < 0.001, as shown in Table 4.

**Table 4:** Comparing IL-1β and IL-6 serum levels among different vaccination statuses of the studied COVID-19 patients.

	Vaccination status	IL-1β	P- value	IL-6	P-value
Partially vaccinated. N = 20	Range	338.8− 943.9	< 0.001	15.06− 64	< 0.001
	Mean ± SEM	556.1 ± 41.9		32.1 ± 3.4	
Fully vaccinated. N = 14	Range	246c338.6		1. 5− 14.9	
	Mean ± SEM	291.7 ± 9.5		6.6 ± 1.4	
Booster dose vaccinated. N = 11	Range	131.05− 245.3		0.18− 1.45	
	Mean ± SEM	199.2 ± 10.6		0.75 ± 0.16	

Significant level at P value < 0.05.

**Fig. 6:**
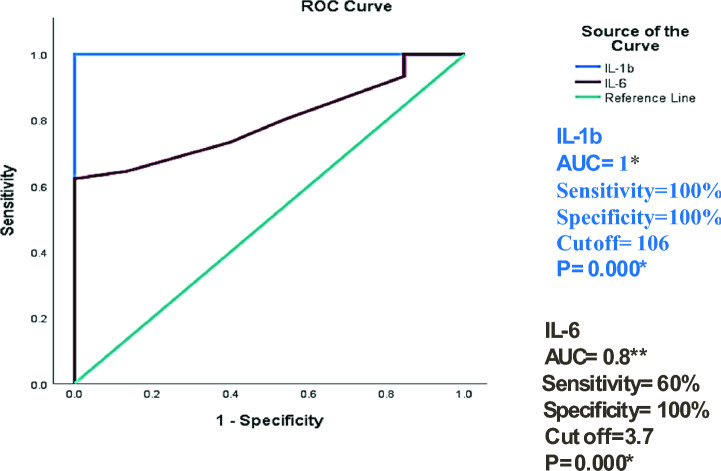
ROC curve analysis for the accuracy of the association between increased level of IL-1β and IL-6 serum levels and COVID-19 infection. AUC, Area under the curve. *AUC (0.9–1) = excellent discriminating efficiency, **AUC (0.7–0.8) = fair discriminating efficiency. *Significant level at P value < 0.05.

The accuracy of the association between increased levels of IL-1β and IL-6 serum levels and COVID-19 infection was assessed by ROC curve analysis. Results revealed that elevated serum levels of the studied cytokines can be used as a diagnostic biomarker with excellent discriminating efficiency for increased IL-1β serum levels (AUC = 1 and P-value = 0.000) and good discriminating efficiency for increased IL-6 serum levels (AUC = 0.8 and P-value = 0.000) as shown in Fig. 6.

### The correlation between IL-1β and IL-6 serum levels with different studied parameters

The correlation between IL-1β and IL-6 serum levels with different parameters was assessed using the Pearson Correlation test, as shown in Table 5. A moderate positive significant correlation between the studied cytokines and lymphocytic count was reported (r = 0.583 and 0.580, respectively, and P-value was < 0.001 for both relations). Additionally, IL-1β levels in COVID-19 cases showed a moderate positive correlation with CRP (r = 0.426) and a strong positive correlation with D-dimer levels (r = 0.916), both statistically significant with P < 0.001. However, studying the correlation between IL-6 and CRP and D. dimer levels in COVID-19 cases showed a significantly strong and moderate positive correlation (r = 0.456 and r = 0.941, respectively, and P-value < 0.001 for both relations).

**Table 5:** Correlation between IL-1β and IL-6 with different studied parameters in COVID-10 patients.

The studied parameter	IL-1β		IL-6	
	R	P-value	R	P-value
Platelet count	− 0.483**	** < 0.001**	-0.553**	** < 0.001**
TLC	0.76*	** < 0.001**	0.765*	** < 0.001**
Lymphocytic count	0.583**	** < 0.001**	0.580**	** < 0.001**
Neutrophil count	0.764*	** < 0.001**	0.79*	** < 0.001**
Monocyte count	0.745*	**0.029**	0.77*	** < 0.001**
D-dimer serum level	0.426**	**0.004**	0.456**	**0.002**
CRP serum level	0.916*	** < 0.001**	0.941*	** < 0.001**
CD4^+^ T-cell count	0.32**	**0.032**	0.283***	**0.060**
PD-1% on CD4^+^ T-cell	0.644**	** < 0.001**	0.698**	** < 0.001**
CD8^+^ T-cell count	0.558**	** < 0.001**	0.577**	** < 0.001**
PD-1% on CD8^+^ T-cell	0.219***	0.148	0.225***	0.137

-Significant level at P value < 0.05.

*r (0.7–1) = strong correlation.

** r (0.3–0.7) = moderate correlation.

***r (< 0.3) = weak correlation.

Significant values are in [bold].

### Relative quantitative expression of serum miRNA-146a and miRNA-133a genes

Analyzing serum miRNA expression levels in the case group compared to healthy controls (which was normalized to 1) using the Mann–Whitney U-test revealed that miR-146a was downregulated in 30 cases (66.7%) and upregulated in 15 cases (33.3%) out of the studied 45 COVID-19 patients with a mean ± SEM of 0.69 ± 0.15. In contrast, miR-133a was upregulated in all 45 COVID-19 patients (mean ± SEM = 503.9 ± 92.54) compared to controls, with a significant difference in the relative fold change (P < 0.001, for each). Detecting the correlation between the expressions of the miRNAs studied in COVID-19 patients versus healthy controls revealed a significantly weak negative correlation between miR-146a expression and COVID-19 disease (r = − 0.214). However, a significant moderate positive correlation between miR-133a expression and COVID-19 disease (r = 0.5) was detected.

The expression of miR-146a varied significantly with disease severity (P < 0.001); among the mild to moderate cases, 14 out of 25 (56%) showed upregulation, while 11 cases (44%) showed downregulation. In contrast, all 20 cases (100%) of the severe to critical cases group were downregulated regarding miR-146a expression. Regarding miR133a expression, it was upregulated in all COVID-19 cases with significant differences in mean and distribution among the mild to moderate group and the severe to critical group Table 6. Detecting the correlation between the expression of the studied miRNAs and the disease severity revealed a significant moderate negative correlation between miR-146a expression and COVID-19 disease severity (r = -0.57). However, there was a significantly strong positive correlation between miR-133a expression and COVID-19 disease severity (r = 0.73), Table 6.

**Table 6:** The difference in expression of miR-146a and miR-133a among different degrees of disease severity cases:

		MiRNA	Mild to moderate cases N = 25	Severe to critical cases N = 20	P -value	R
miRNA-146a	Total	Range	0.2—5.3	0.001—0.144	< 0.001	− 0.57
		Mean ± SEM	1.2 ± 0.2	0.04 ± 0.01		
	Upregulated cases	N (%)	11 (44%)	0 (0%)	< 0.001	
		Range	1.2 – 5.3	-		
		Mean ± SEM	2.1 ± 0.3	-		
	Downregulated cases	N (%)	14 (56%)	20 (100%)		
		Range	0.2–0.9	0.001—0.144		
		Mean ± SEM	0.48 ± 0.05	0.04 ± 0.01		
miRNA-133a	Total	Range	1.9—374.6	431.2—2290.2	**< 0.001**	0.73
		Mean ± SEM	99.5 ± 21.3	1009.4 ± 140.1		
	Upregulated cases	N (%)	25 (100%)	20 (100%)	NA	
	Downregulated cases	N (%)	0 (0%)	0 (0%)		

NA = Not Applicable, as there is no statistical analysis done for the regulation of expression of miR-133a among different degrees of disease severity as its upregulation result is a constant in all cases included in this study.

Significant values are in [bold].

**Fig. 7:**
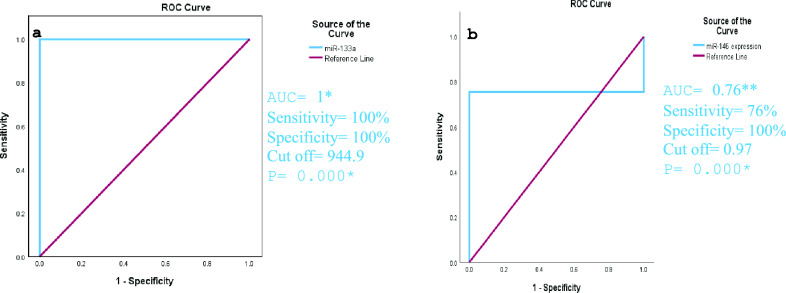
(**a**) ROC curve analysis for the accuracy of the association between upregulation of miR-133a and COVID-19 infection. (**b**) ROC curve analysis for the accuracy of the association between downregulation of miR-146a and COVID-19 infection. AUC, Area under the curve. *AUC (0.9–1) = excellent discriminating efficiency, **AUC (0.7–0.8) = fair discriminating efficiency. *Significant level at P value < 0.05.

The accuracy of the association between the upregulation of miR-133a and the downregulation of miR-146a and COVID-19 infection was done by ROC curve analysis. It revealed that miR-133a upregulation had excellent discriminating efficiency (AUC = 1 and P-value 0.000), while downregulation of miR-146a showed fair discriminating efficiency (AUC = 0.76 and P-value 0.000) as shown in Fig. 7.

### The correlation between miR-133a and miR-146a expression with different studied parameters

The correlation between IL-1β and IL-6 serum levels and miR-146a expression in COVID-19 cases was assessed using the Pearson Correlation test, as shown in Table 7. A significant moderate negative correlation was found between the studied cytokines and miR-146a (r = − 0.599 and r = − 0.562, respectively, and P-value < 0.001 for both relations). Studying the correlation between IL-1β and IL-6 serum levels and miR-133a expression in COVID-19 cases was found to give a significantly strong positive correlation between the studied cytokines and miR-133a (r = − 0.979 and r = − 0.975, respectively, and P-value < 0.001 for both relations) as shown in Table 7.

**Table 7:** Correlation between expression levels of serum miR-133a and miR-164a and different studied parameters in COVID-19 patients.

The studied parameter	miR-133a		miR-146a	
	R	P-value	R	P-value
Platelet count	− 0.49**	< 0.001	0.25***	0.094
TLC	0.7*	** < 0.001**	− 0.65**	** < 0.001**
Lymphocytic count	0.54**	** < 0.001**	− 0.55**	** < 0.001**
Neutrophil count	0.7*	** < 0.001**	− 0.65**	**0.02**
Monocyte count	0.7*	** < 0.001**	− 0.57**	** < 0.001**
D-dimer serum level	0.459**	**0.002**	− 0.303**	**0.043**
CRP serum level	0.89*	** < 0.001**	− 0.64**	** < 0.001**
CD4^+^ T-cell count	0.27***	**0.072**	− 0.41**	**0.006**
PD-1% on CD4^+^ T-cell	0.63**	** < 0.001**	− 0.41**	**0.006**
CD8^+^ T-cell count	0.52**	** < 0.001**	− 0.420**	**0.004**
PD-1% on CD8^+^ T-cell	0.14***	**0.374**	− 0.26***	**0.085**
IL-1β serum level	0.979*	** < 0.001**	− 0.599**	** < 0.001**
IL-6 serum level	0.975*	** < 0.001**	− 0.57**	** < 0.001**

-Significant level at P value < 0.05.

*r (0.7–1) = strong correlation.

** r (0.3–0.7) = moderate correlation.

***r (< 0.3) = weak correlation.

### Regression analysis

Binary logistic regression analysis of CD4 + and CD8 + T-cell counts, PD-1 expression on CD4 + and CD8 + T-cells, and the CD4 + :CD8 + ratio revealed significant differences between the COVID-19 patient group and the control group. COVID-19 patients showed a significant decrease in CD4 + and CD8 + T-cell counts, with an increase in the percentage of PD-1 on both CD4 + and CD8 + T-cells, and a higher CD4 + :CD8 + ratio. These findings had strong predictive values, as the Odds Ratio (OR) was above one. Also, binary logistic regression analysis for the CD4^+^ and CD8^+^ T-cell count, PD-1 on CD4^+^ and CD8^+^ T-cells, and CD4^+^:CD8^+^ ratio was done among varying disease severity groups. It showed a significant increase in CD4^+^ and CD8^+^ T-cell count, increase in the percentage of PD-1 on CD4^+^ and CD8^+^ T-cells, and a decreased CD4^+^:CD8^**+**^ ratio in severe to critical patients compared to mild to moderate patients. These findings had accurate, significant predictive values, as the Odds Ratio (OR) was above one, as shown in Table 8.

**Table 8:** Binary logistic regression analysis for the studied parameters in cases to control groups and in different degrees of disease severity.

Parameters	Cases to control		Degree of severity	
	Odds ratio	P value	Odds ratio	P value
CD4^+^ T-cell count	1.01	0.001*	1.01	0.001*
PD-1% on CD4^+^	1.02	0.001*	1.01	0.001*
CD8^+^ T-cell count	1.05	0.001*	1.02	0.001*
PD-1% on CD8^+^	1.2	0.001*	1.01	0.001*
CD4^+^:CD8^+^ ratio	1.05	0.001*	5	0.001*
IL-1β	1.3	0.001*	1.26	0.001*
IL-6	1.2	0.001*	5.7	0.001*
miR-133a	1.07	0.001*	1.2	0.001*
miR-146a	373.9	0.001*	333.3	0.001*

*Significant level at P value < 0.05.

A significant increase in the serum levels of IL-1β and IL-6 in COVID-19 patients compared to controls was assessed by binary logistic regression with accurate, significant predictive values, as the Odds Ratio was 1.3 and 1.2, respectively**.** In addition, when comparing the mild to moderate group with the severe to critical group, there was a significant increase in IL-1β and IL-6 levels in the severe to critical patients, with Odds Ratios of 1.26 and 5.7, respectively, demonstrating accurate and significant predictive values, as shown in Table 8.

Regarding the expression of miR-133a and miR-146a, binary logistic regression showed a significant increase in expression of miR-133a and a decrease in expression of miR-146a in COVID-19 patients compared to controls, with accurate significant predictive values, as the Odds Ratio was 1.1 and 373.9, respectively. However, a significant increase in expression of miR-133a and a decrease in expression of miR-146a in severe to critical patients compared to mild to moderate patients were detected with accurate, significant predictive values, as the Odds Ratio was 1.2 and 333, respectively, as shown in Table 8.

## Discussion

COVID-19 is a serious infectious pandemic; thus, it is of great significance to study the predictors of disease severity that will help guide case management. In this study, some demographics and laboratory data of COVID-19 patients with different degrees of disease severity were compared within each group and with healthy controls. In addition, the detection of CD4 + and CD8 + T-cells and PD-1 exhaustion markers on T-lymphocyte subsets was done by flow cytometry.

The current study found that most of patients in the severe to critical group were older than those in the mild to moderate group. These findings are consistent with several previous studies^17–19^. This could be due to an age-dependent decline in the immune function of the body, leading to reduced protection against COVID-19^20^*.*

According to our study, smoking is one of the risk factors that increase the severity of COVID-19 symptoms. This finding is consistent with another study that showed that smokers are twice as likely to experience severe or critical COVID-19 symptoms compared to non-smokers^21^. This study showed that the progression of the disease is influenced by the place of residence, where 80% of severe to critical cases were from rural areas, while 84% of mild to moderate cases were from urban areas. These findings were supported by another study, which revealed that almost 50% of rural residents are at a high risk of being hospitalized and experiencing severe illness if they contract COVID-19^22^.

It was previously reported that vaccination reduces symptomatic COVID-19 infections and provides greater protection against severe disease^23^. In our study, all the severe to critical patients were partially vaccinated (100%). At the same time, no one in the mild to moderate group was unvaccinated or partially vaccinated. However, 56% of cases in the mild to moderate group were fully vaccinated, and 44% had their booster dose vaccination. These findings align with another study that observed that the proportion of fully vaccinated patients who developed critical disease was significantly lower, and fully vaccinated patients also had significantly shorter mean length of hospital stay^24^. In this study, fever was the most frequent clinical presentation among COVID-19 patients; 86.7% of all studied cases had a fever, and only 6 (24%) of mild to moderate cases had no fever. Also, other studies reported that the most common COVID-19 presentation was fever^25–27^. It was revealed in this study that low oxygen saturation and high respiratory rate increased the likelihood of severity and critical consequences, which agrees with other previous findings^28–30^.

In this study, no abnormalities in platelet counts were observed in the mild to moderate group patients, while 40% of the severe to critical group showed thrombocytopenia. This was in line with another study that reported thrombocytopenia more frequently among severe cases than mild cases^31^**.** Immune thrombocytopenia (ITP) has been reported in COVID-19 patients, whose mechanism is unclear, however, it may be attributed to the underlying immune dysregulation in COVID-19, leading to the production of antiplatelet antibodies that destroy circulating platelets and megakaryocytes in the bone marrow^32^**.**

Regarding lymphopenia studied among COVID-19 patients in our study, most patients (88.9%) had a decrease in lymphocyte levels, with no significant difference among different degrees of severity. This was similar to a study that surveyed 1,099 patients and found that 83.2% of the patients with COVID-19 had a decreased lymphocyte count at the time of hospital admission^33^**.** Although it is still unclear what the underlying mechanism for lymphopenia in COVID-19 patients is, several authors relate it to increased apoptosis, cytokine-induced lymphocyte inhibition or down-regulation, metabolic disorders, and elevated glucocorticoid levels. The results of the current study indicated a significant reduction in the levels of CD3^+^CD4^+^ and CD3^+^CD8^+^ T cells in the case group compared to the healthy group and in the severe to critical group compared to the mild to moderate group, suggesting the decrease in CD4^+^ and CD8^+^ T-cell counts as an independent prognostic factor for the disease. These findings agree with what was observed by previous studies reporting the association of disease severity with a decreased count of CD4^+^ T cells or CD8^+^ T cells or both CD4^+^ and CD8^+^ T-cell counts^36–38^. The current study showed that the percentage of PD-1 expressed on CD3^+^CD4^+^ T cells and CD3^+^CD8^+^ T cells was significantly increased in cases compared to the control group and in the severe to critical group compared to the mild to moderate group. Our results suggested that the increased expression of PD-1 on CD4^+^ T cells may be used as a diagnostic biomarker with excellent discriminating efficiency between cases and control groups. However, the increased expression of PD-1 on CD8^+^ T-cells showed a fair discriminating efficiency, proposing the increase in the percentage of PD-1 on CD4^+^ and CD8^+^ T-cells in COVID-19 patients to be independent prognostic factors for the disease. These results agree with many previous studies^35,39,40^**.** Significant reductions in T cell populations and the simultaneous overexpression of PD-1 on CD4+ and CD8+ T cells in severe cases of COVID-19 were also confirmed by other studies^41,42^***.*** Our findings suggest that an increased expression of PD-1 in CD4+ and CD8+ T cells indicates functional exhaustion of T cells, determined by a combination of the decrease in T cell counts and the overexpression of PD-1. This has been confirmed by a study that characterized a wide immunological profile, including additional immune checkpoints such as TIM-3 and NKG2A in addition to PD-1^44^**.** Abnormal coagulation function, including elevated D-dimer, has been demonstrated to be involved in the disease progression of COVID-19. In this study, we analyzed the association between elevated D-dimer levels and the disease severity of COVID-19 and found that the level of D-dimer was markedly increased in severe to critical patients, as reported in previous studies^46,47^.

In this study, all cases had elevated CRP levels, giving a strong positive significant correlation between increased CRP levels and COVID-19 disease severity; this result aligns with other studies^48–50^. Therefore, CRP may be a suitable marker for assessing a patient’s condition with other clinical findings. Elevated levels of CRP might be linked to the overproduction of inflammatory cytokines in severe patients with COVID‐19. Consequently, the IL-1/IL-6/CRP axis is essential for developing inflammation^51^. Moreover, liver cells release CRP in response to IL-6 stimulation. Therefore, CRP levels can be used as a surrogate marker due to the correlation between CRP levels and IL-6. According to recent studies, it was strikingly shown that the level of inflammatory cytokines increased in COVID-19^52^. This study detected the serum level of two inflammatory cytokines, IL-1β and IL-6, and results showed a significant elevation of their serum levels in COVID-19 patients compared to the healthy group and in the severe to critical group compared to the mild to moderate group. These findings suggest that IL-1β is a potential marker with excellent discriminating efficiency to assess COVID-19 infection, and IL-6 is a potential marker with fair discriminating efficiency to assess COVID-19 infection. These results agree with previous studies that linked the increase of IL-1β and IL-6 serum levels with the severity of COVID-19 infection^53,54^. According to a systematic review and meta-analyses of 10 cohort studies, including 1798 patients, elevated levels of IL-6 were observed in patients with COVID-19^55^. Our study showed that the serum level of IL-6 was 32.1 ± 15.59 pg./mL in patients in the severe to the critical group, this is much lower than what is reported by Rostamiana et al. that reported that the serum level of IL-6 was 517 ± 796 pg/mL in patients with severe disease^56^. This lower level of IL-6 in our study may be attributed to steroid therapy taken by the patients, where steroids and other anti-inflammatory drugs are the treatment of choice to inhibit the development of the cytokine storm^57^. It may also be explained by the effect of the vaccination taken by the patients under study. Our results showed that the increase in IL-1β and IL-6 serum levels was positively correlated with CRP and D-dimer serum levels. These results agree with another Egyptian study that found a significant difference in D-dimer and IL-1β serum levels between COVID-19 patients with different degrees of disease severity and reported that they were positively correlated with each other and with the disease severity^58^. Other studies have also indicated that IL-1 and IL-6, along with other inflammatory markers, may play critical roles in thrombin and fibrin generation by modulating the coagulation system in COVID-19 patients^59,60^. This can be explained by the role of IL-1 and IL-6 in coagulopathy, as it potentially induces coagulation factors, including thrombin factor, fibrinogen, and factor VIII^61^.

Different miRNAs are altered during COVID-19 infection. Alterations in miRNA levels have been linked to the severity of COVID-19, particularly in cases suffering from comorbid conditions. In the current study, the expression levels of two circulating miRNAs (miR-133a and miR-146a) were assessed in COVID-19 patients and healthy controls. The results showed a decrease in miR-146a expression in COVID-19 patients compared to healthy controls, suggesting it is a potential marker with fair discriminating efficiency to assess COVID-19 infection. This decrease was associated with the severity of the disease, with accurate and significant predictive values for the severity of the disease. This agrees with the results reported by previous studies^8,62–64^. A previous study suggested that miR-146a plays a crucial role as a regulator of viral infection by playing a significant role in inflammatory settings and in the T-lymphocyte-mediated adaptive immune response, which has a pivotal role in viral infections^14^. It has been found that miR-146a has a negative effect on NF-κB, which is a well-known transcription factor of the IL-6 gene. COVID-19 patients have shown increased levels of IL-6 and reduced levels of miR-146a when compared to healthy individuals of the same age. This suggests an imbalance in the IL-6/miR-146a physiological axis, which could be involved in the development of SARS-CoV-2 infection^65^. As regards miR-133a, the results showed a significant increase in miR-133a in COVID-19 patients compared to healthy controls prosing it as a potential marker with excellent discriminating efficiency in the assessment of COVID-19 infection, with high sensitivity and specificity. This increase was found to be associated with the severity of the disease, which is consistent with previous studies that reported that miR-133a levels were higher in COVID-19 patients than in normal controls^9^ and among severe COVID-19 cases compared to mild cases, predicting poor survival with its overexpression^66^. The miR-133a is elevated in sepsis patients and predicts mortality in critically ill patients. In critically ill COVID-19 patients without sepsis, miR-133a levels were highest in those with cardiopulmonary diseases. miR-133a is elevated in sepsis patients and predicts mortality in critically ill patients^9^. Our results showed that the significant increase in expression of miR-133a in COVID-19 patients compared to controls has accurate and significant predictive values, and the expression of miR-133a in the severe to critical group compared to the mild to moderate group has accurate and significant predictive values for the severity of the disease. Unfortunately, due to limited funding and budget constraints, it was not feasible for our research team to use different methods for each parameter of the predictors studied. Because of this, we chose the most used and widely accepted method for each parameter based on previous studies in literature. Also, we could not further investigate more parameters helpful for diagnosis and assessment of COVID-19 disease severity.

## Conclusion

We conclude that T cells decreased in patients with COVID-19 with elevation of the CD4:CD8 ratio compared to the healthy control group and the ratios were higher in the mild to moderate group than in the severe to critical group, suggesting it is an independent prognostic factor for the disease, which was significantly associated with disease severity. Also, it was concluded that the percentage of PD-1 expressed on T-cells was significantly increased in cases compared to the control group, indicating the exhaustion of T-cells in COVID-19 infection. Hence, the significant role of immunological modulation in COVID-19 infection involves the exhaustion of immune cells and the overactivation of the immune response, contributing to the severity of the disease. Taken together, our data highlights the changes in cytokine secretion in serum and miRNA expression, which are significantly affected by COVID-19 infection. Further studies on more T cell exhaustion markers, more significantly identified cytokines and miRNAs with large sample sizes from different regions are recommended to describe other potential markers of COVID-19 disease severity and to understand the interaction between SARS-CoV-2 and the immune system, which could aid in developing novel immune-based intervention strategies. Thus, COVID-19 immunotherapy will be more targeted and effective.

## Supplementary Information

Supplementary Information.

## Data Availability

All data generated or analyzed during this study are included in this article.

## References

[1] Zekri, A. N. et al. Genome sequencing of SARS-CoV-2 in a cohort of Egyptian patients revealed mutation hotspots that are related to clinical outcomes. *Biochim. Biophys. Acta* **1867**(8), 166154 (2021). 10.1016/j.bbadis.2021.166154 PMC8079944 33932525

[2] Bal, A., Agrawal, R., Vaideeswar, P., Arava, S. & Jain, A. COVID-19: An up-to-date review—From morphology to pathogenesis. *Indian J. Pathol. Microbiol.* **63**(3), 358–366 (2020). 32769322 10.4103/IJPM.IJPM_779_20

[3] Henderson LA, Canna SW, Schulert GS, Volpi S, Lee PY, Kernan KF, Caricchio R, Mahmud S, Hazen MM, Halyabar OJA *et al*: On the alert for cytokine storm: immunopathology in COVID‐19. 2020, 72(7):1059–1063. 10.1002/art.41285 PMC7262347 32293098

[4] Sette, A. & Crotty, S. Adaptive immunity to SARS-CoV-2 and COVID-19. *Cell* **184**(4), 861–880 (2021). 33497610 10.1016/j.cell.2021.01.007 PMC7803150

[5] Detsika, M. G. et al. Combination of the CD8(+):B-cell and neutrophil-to-lymphocyte ratio as a novel prediction model for intubation need and disease severity in COVID-19 patients. *In Vivo* **35**(6), 3305–3313 (2021). 34697162 10.21873/invivo.12626 PMC8627713

[6] Henderson, L. A. et al. On the alert for cytokine storm: Immunopathology in COVID-19. *Arthrit. Rheumatol* **72**(7), 1059–1063 (2020). 10.1002/art.41285 PMC7262347 32293098

[7] Silverio, R., Gonçalves, D. C., Andrade, M. F. & Seelaender, M. Coronavirus disease 2019 (COVID-19) and nutritional status: the missing link?. *Adv. Nutr* **12**(3), 682–692 (2021). 32975565 10.1093/advances/nmaa125 PMC7543263

[8] Sabbatinelli, J. et al. Decreased serum levels of the inflammaging marker miR-146a are associated with clinical non-response to tocilizumab in COVID-19 patients. *Mech. Ageing Dev.* **193**, 111413 (2021). 33307107 10.1016/j.mad.2020.111413 PMC7722494

[9] Gutmann, C. et al. Association of cardiometabolic microRNAs with COVID-19 severity and mortality. *Cardiovasc. Res.* **118**(2), 461–474 (2022). 34755842 10.1093/cvr/cvab338 PMC8689968

[10] Masoud H, Elassal G, Zaky S, Baki A, Ibrahem H, Amin W, Abdelbary A, Abdalmohsen A, Hassany M, Eid A *et al*: Management Protocol for COVID-19 Patients Version 1.4/30th May 2020 Ministry of health and population (MOHP), Egypt. In*.*; 2020.

[11] Li, M. et al. Association of COVID-19 vaccination and clinical severity of patients infected with delta or omicron variants—China, May 21, 2021-February 28, 2022. *China CDC Wkly* **4**(14), 293–297 (2022). 35433093 10.46234/ccdcw2022.074 PMC9008265

[12] Zhang, W. et al. PD-1 expression on the surface of peripheral blood CD4(+) T cell and its association with the prognosis of patients with diffuse large B-cell lymphoma. *Cancer Med.* **5**(11), 3077–3084 (2016). 27709793 10.1002/cam4.874 PMC5119962

[13] Zhang, Y., Zhu, W., Zhang, X., Qu, Q. & Zhang, L. Expression and clinical significance of programmed death-1 on lymphocytes and programmed death ligand-1 on monocytes in the peripheral blood of patients with cervical cancer. *Oncol. Lett.* **14**(6), 7225–7231 (2017). 29344157 10.3892/ol.2017.7105 PMC5754902

[14] Ostrycharz E, Hukowska-Szematowicz B: Micro-players of great significance—Host microRNA signature in viral infections in humans and animals. 2022, 23(18):10536. 10.3390/ijms231810536 PMC9504570 36142450

[15] Chen, X. et al. Characterization of microRNAs in serum: a novel class of biomarkers for diagnosis of cancer and other diseases. *Cell Res.* **18**(10), 997–1006 (2008). 18766170 10.1038/cr.2008.282

[16] McElvany, S. W., Ross, M. M., Goroff, N. S. & Diederich, F. Cyclocarbon coalescence: Mechanisms for tailor-made fullerene formation. *Science* **259**(5101), 1594–1596 (1993). 17733024 10.1126/science.259.5101.1594

[17] Huang, C. et al. Clinical features of patients infected with 2019 novel coronavirus in Wuhan China. *Lancet* **395**(10223), 497–506 (2020). 31986264 10.1016/S0140-6736(20)30183-5 PMC7159299

[18] Al-Kuraishy, H. M. et al. COVID-19 in relation to hyperglycemia and diabetes mellitus. *Front. Cardiovasc. Med.* **8**, 644095 (2021). 34124187 10.3389/fcvm.2021.644095 PMC8189260

[19] Pan, F. et al. Time course of lung changes at chest CT during recovery from coronavirus disease 2019 (COVID-19). *Radiology* **295**(3), 715–721 (2020). 32053470 10.1148/radiol.2020200370 PMC7233367

[20] Zhou, P. et al. A pneumonia outbreak associated with a new coronavirus of probable bat origin. *Nature* **579**(7798), 270–273 (2020). 32015507 10.1038/s41586-020-2012-7 PMC7095418

[21] Reddy, R. K. et al. The effect of smoking on COVID-19 severity: A systematic review and meta-analysis. *J. Med. Virol.* **93**(2), 1045–1056 (2021). 32749705 10.1002/jmv.26389 PMC7436545

[22] Kaufman, B. G., Whitaker, R., Pink, G. & Holmes, G. M. Half of rural residents at high risk of serious illness due to COVID-19, creating stress on rural hospitals. *J Rural Health Off J Am. Rural Health Assoc. Nat. Rural Health Care Assoc* **36**(4), 584–590 (2020). 10.1111/jrh.12481 PMC7361543 32603030

[23] Lopez Bernal, J. et al. Effectiveness of the Pfizer-BioNTech and Oxford-AstraZeneca vaccines on covid-19 related symptoms, hospital admissions, and mortality in older adults in England: test negative case-control study. *BMJ* **373**, n1088 (2021). 33985964 10.1136/bmj.n1088 PMC8116636

[24] Sezen, Y. I. et al. Risk factors and the impact of vaccination on mortality in COVID-19 patients. *Bratisl. Lek. Listy* **123**(6), 440–443 (2022). 35576546 10.4149/BLL_2022_068

[25] Ghweil, A. A. et al. Characteristics, outcomes and indicators of severity for COVID-19 among sample of ESNA quarantine hospital’s patients, Egypt: A retrospective study. *Infect. Drug Resist.* **13**, 2375–2383 (2020). 32765012 10.2147/IDR.S263489 PMC7381791

[26] Chan, J. F. et al. A familial cluster of pneumonia associated with the 2019 novel coronavirus indicating person-to-person transmission: a study of a family cluster. *Lancet* **395**(10223), 514–523 (2020). 31986261 10.1016/S0140-6736(20)30154-9 PMC7159286

[27] Wang, F. et al. Characteristics of peripheral lymphocyte subset alteration in COVID-19 pneumonia. *J. Infect. Dis.* **221**(11), 1762–1769 (2020). 32227123 10.1093/infdis/jiaa150 PMC7184346

[28] Charoenngam, N., Alexanian, S. M., Apovian, C. M. & Holick, M. F. Association between hyperglycemia at hospital presentation and hospital outcomes in COVID-19 patients with and without type 2 diabetes: A retrospective cohort study of hospitalized inner-city COVID-19 patients. *Nutrients* **13**(7), 2199 (2021). 34206813 10.3390/nu13072199 PMC8308462

[29] Ramatillah, D. L. & Isnaini, S. Treatment profiles and clinical outcomes of COVID-19 patients at private hospital in Jakarta. *PLoS ONE* **16**(4), e0250147 (2021). 33861777 10.1371/journal.pone.0250147 PMC8051784

[30] Fathalla, L. A. et al. Laboratory biomarker predictors for disease progression and outcome among Egyptian COVID-19 patients. *Int. J. Immunopathol. Pharmacol.* **22**(36), 03946320221096207 (2022). 10.1177/03946320221096207 PMC9150244 35622504

[31] Mocan M, Chiorescu RM, Tirnovan A, Buksa BS, Farcaș ADJJoCM: Severe thrombocytopenia as a manifestation of COVID-19 infection. 2022, 11(4):1088. 10.3390/jcm11041088 PMC8877916 35207365

[32] Davoodian A, Umeh C, Novatcheva E, Sassi GP, Ahaneku H, Kundu A, Novatcheva ED. Severe immune thrombocytopenia post-COVID-19: a case report. Cureus. 2021 Nov 13;13(11). 10.7759/cureus.19544 PMC8668258 34934562

[33] Guan, W. J. et al. Clinical characteristics of coronavirus disease 2019 in China. *N. Engl. J. Med.* **382**(18), 1708–1720 (2020). 32109013 10.1056/NEJMoa2002032 PMC7092819

[34] Danwang, C. et al. A meta-analysis of potential biomarkers associated with severity of coronavirus disease 2019 (COVID-19). *Biomark. Res.* **8**(1), 37 (2020). 32879731 10.1186/s40364-020-00217-0 PMC7456766

[35] Bellesi, S. et al. Increased CD95 (Fas) and PD-1 expression in peripheral blood T lymphocytes in COVID-19 patients. *Br. J. Haematol.* **191**(2), 207–211 (2020). 32679621 10.1111/bjh.17034 PMC7405050

[36] Aljabr, W. et al. Evaluation of the levels of peripheral CD3+, CD4+, and CD8+ T cells and IgG and IgM antibodies in COVID-19 patients at different stages of infection. *Microbiol. Spect.* **10**(1), e00845-e921 (2022). 10.1128/spectrum.00845-21 PMC8865559 35196808

[37] Chong VC, Lim KGE, Fan BE, Chan SS, Ong KH, Kuperan PJBJoH: Reactive lymphocytes in patients with Covid‐19. 2020, **189**(5):844. 10.1111/bjh.16690 PMC7262365 32297330

[38] Jimeno, S. et al. Prognostic implications of neutrophil-lymphocyte ratio in COVID-19. *Eur. J. Clin. Invest.* **51**(1), e13404 (2021). 32918295 10.1111/eci.13404

[39] Mallajosyula, V. et al. CD8+ T cells specific for conserved coronavirus epitopes correlate with milder disease in patients with COVID-19. *Sci. Immunol.* **6**(61), 5669 (2021). 10.1126/sciimmunol.abg5669 PMC8975171 34210785

[40] Liana, P. et al. CD4+ and CD8+ cell counts are significantly correlated with absolute lymphocyte count in hospitalized COVID-19 patients: A retrospective study. *PeerJ* **23**(11), e15509 (2023). 10.7717/peerj.15509 PMC10292192 37377785

[41] Diao, B. et al. Reduction and functional exhaustion of T cells in patients with coronavirus disease 2019 (COVID-19). *Front. Immunol.* **1**(11), 544639 (2020). 10.3389/fimmu.2020.00827 PMC7205903 32425950

[42] Lei, C. et al. Wang JJJoCV: Factors associated with clinical outcomes in patients with coronavirus disease 2019 in Guangzhou. *China.* **133**, 104661 (2020). 10.1016/j.jcv.2020.104661 PMC7554493 33096290

[43] Beserra, D. R. et al. Upregulation of PD-1 expression and high sPD-L1 levels associated with COVID-19 severity. *J. Immunol. Res.* **2022**(1), 9764002 (2022). 35971391 10.1155/2022/9764002 PMC9375698

[44] de Candia, P., Prattichizzo, F., Garavelli, S. & Matarese, G. T cells: Warriors of SARS-CoV-2 infection. *Trends Immunol.* **42**(1), 18–30 (2021). 33277181 10.1016/j.it.2020.11.002 PMC7664351

[45] Varchetta, S. et al. Unique immunological profile in patients with COVID-19. *Cell. Mol. Immunol.* **18**(3), 604–612 (2021). 33060840 10.1038/s41423-020-00557-9 PMC7557230

[46] Yu, H. H., Qin, C., Chen, M., Wang, W. & Tian, D. S. D-dimer level is associated with the severity of COVID-19. *Thromb. Res.* **195**, 219–225 (2020). 32777639 10.1016/j.thromres.2020.07.047 PMC7384402

[47] Han, H. et al. Prominent changes in blood coagulation of patients with SARS-CoV-2 infection. *Clin. Chem. Lab. Med.* **58**(7), 1116–1120 (2020). 32172226 10.1515/cclm-2020-0188

[48] Chen, L., Li, X., Chen, M., Feng, Y. & Xiong, C. The ACE2 expression in human heart indicates new potential mechanism of heart injury among patients infected with SARS-CoV-2. *Cardiovasc. Res.* **116**(6), 1097–1100 (2020). 32227090 10.1093/cvr/cvaa078 PMC7184507

[49] Ali, N. Elevated level of C-reactive protein may be an early marker to predict risk for severity of COVID-19. *J. Med. Virol.* **92**(11), 2409–2411 (2020). 32516845 10.1002/jmv.26097 PMC7301027

[50] Yao, X. H. et al. A pathological report of three COVID-19 cases by minimal invasive autopsies. *Zhonghua bing li xue za zhi Chinese journal of pathology* **49**(5), 411–417 (2020). 32172546 10.3760/cma.j.cn112151-20200312-00193

[51] Zhu, X. et al. Dynamics of inflammatory responses after SARS-CoV-2 infection by vaccination status in the USA: A prospective cohort study. *The Lancet Microbe* **4**(9), e692–e703 (2023). 37659419 10.1016/S2666-5247(23)00171-4 PMC10475695

[52] Hirano, T. & Murakami, M. COVID-19: A new virus, but a familiar receptor and cytokine release syndrome. *Immunity* **52**(5), 731–733 (2020). 32325025 10.1016/j.immuni.2020.04.003 PMC7175868

[53] Montazersaheb, S. et al. COVID-19 infection: An overview on cytokine storm and related interventions. *Virol. J.* **19**(1), 92 (2022). 35619180 10.1186/s12985-022-01814-1 PMC9134144

[54] Sarhan WM, Shalaby SM, Zidan N, Elhawary A, Ismail N, Makani V, Abd elnour HM: Cytokine Storm in COVID-19 Patients: Association between cytokines and disease severity %J Zagazig Univ. Med. J. 2021, 27(6):1640–1653.

[55] Coomes, E. A. & Haghbayan, H. Interleukin-6 in Covid-19: A systematic review and meta-analysis. *Rev. Med. Virol.* **30**(6), 1–9 (2020). 32845568 10.1002/rmv.2141 PMC7460877

[56] Rostamian A, Ghazanfari T, Arabkheradmand J, Edalatifard M, Ghaffarpour S, Salehi M, Raeeskarami S-R, Aliabadi M, Chenary M, Mirsharif E *et al*: Interleukin-6 as a Potential Predictor of COVID-19 Disease Severity in Hospitalized Patients and its Association with Clinical Laboratory Routine Tests. *Immunoregulation* 2020:29–36.

[57] Keewan, E., Beg, S. & Naser, S. A. Anti-TNF-α agents Modulate SARS-CoV-2 Receptors and Increase the Risk of Infection Through Notch-1 Signaling. *Front. Immunol.* **12**, 641295 (2021). 34025650 10.3389/fimmu.2021.641295 PMC8134694

[58] Abd El-Ghani, S. E. S. et al. Serum interleukin 1β and sP-selectin as biomarkers of inflammation and thrombosis, could they be predictors of disease severity in COVID 19 Egyptian patients?(a cross-sectional study). *Thromb. J.* **20**(1), 77 (2022). 36522776 10.1186/s12959-022-00428-5 PMC9754776

[59] Ahmad, F., Kannan, M. & Ansari, A. W. Role of SARS-CoV-2 -induced cytokines and growth factors in coagulopathy and thromboembolism. *Cytokine Growth Factor Rev.* **63**, 58–68 (2022). 34750061 10.1016/j.cytogfr.2021.10.007 PMC8541834

[60] Carsana, L. et al. Pulmonary post-mortem findings in a series of COVID-19 cases from northern Italy: A two-centre descriptive study. *Lancet. Infect. Dis* **20**(10), 1135–1140 (2020). 32526193 10.1016/S1473-3099(20)30434-5 PMC7279758

[61] Kang, S. & Kishimoto, T. Interplay between interleukin-6 signaling and the vascular endothelium in cytokine storms. *Exp. Mol. Med.* **53**(7), 1116–1123 (2021). 34253862 10.1038/s12276-021-00649-0 PMC8273570

[62] Giannella, A. et al. Circulating microRNA signatures associated with disease severity and outcome in COVID-19 patients. *Front. Immunol.* **13**, 968991 (2022). 36032130 10.3389/fimmu.2022.968991 PMC9403711

[63] Grant, M. C. et al. The prevalence of symptoms in 24,410 adults infected by the novel coronavirus (SARS-CoV-2; COVID-19): A systematic review and meta-analysis of 148 studies from 9 countries. *PLoS ONE* **15**(6), e0234765 (2020). 32574165 10.1371/journal.pone.0234765 PMC7310678

[64] Martínez-Fleta, P. et al. A differential signature of circulating miRNAs and cytokines between COVID-19 and community-acquired pneumonia uncovers novel physiopathological mechanisms of COVID-19. *Front. Immunol.* **12**, 815651 (2021). 35087533 10.3389/fimmu.2021.815651 PMC8787267

[65] Sun, K., Gu, L., Ma, L. & Duan, Y. Atlas of ACE2 gene expression reveals novel insights into transmission of SARS-CoV-2. *Heliyon* **7**(1), e05850 (2021). 33392409 10.1016/j.heliyon.2020.e05850 PMC7762714

[66] Filbin, M. R. et al. Longitudinal proteomic analysis of severe COVID-19 reveals survival-associated signatures, tissue-specific cell death, and cell-cell interactions. *Cell Rep. Med.* **2**(5), 100287 (2021). 33969320 10.1016/j.xcrm.2021.100287 PMC8091031

