# Supplementary material for: Potential predictors of COVID-19 disease infection and severity in Egypt
Source: Sci Rep. 2026 Jan 29;16:4093. doi: 10.1038/s41598-025-34444-y (PMC12855883; doi:10.1038/s41598-025-34444-y)

**Scientific reports**

**Potential predictors of COVID-19 disease infection and severity in Egypt**

**Wedad M. Abdelraheem^1^, Raghda Raouf Shady^1^, Wafaa K.M Mahdi^1^, Mohamed Ibrahim Bassyouni^1^, Heba S. Kamel^2^, Yosra M. Mousa^3^, Shiamaa F. Kamel^4^,** **Manal Mohamed Saber^5^, Soha S. Abdelrahim^1^**

**Supplementary Table S1: PCR primers used for relative quantification of the targeted genes: miR-146a and miR-133a, and the reference gene miR-16.**

| The gene | Forward primer sequence | Reverse primer sequence |
| --- | --- | --- |
| *miR-146a* | ACACTCCAGCTGGGTGAGAACTGAATTCCA | CTCAACTGGTGTCGTGGAGTCGGCAATTCAGTTGAGAACCCATG |
| *miR-133a* | ACACTCCAGCTGGGTTGGTCCCCTTCAACC | CTCAACTGGTGTCGTGGAGTCGGCAATTCAGTTGAGACAGCTGG |
| *miR-16* | ACACTCCAGCTGGGTAGCAGCACGTAAATA | CTCAACTGGTGTCGTGGAGTCGGCAATTCAGTTGAGCGCCAATA |

**Supplementary Table S2: Comparison of demographic data in COVID-19 patients among different degrees of disease severity.**

| **Data** | | **Total patients (N=45)** | **Mild to Moderate (N=25)** | **Severe to Critical (N=20)** | | **P-value** |
| --- | --- | --- | --- | --- | --- | --- |
|  |  | **N (%)** | | | |  |
| **Age in years** | **Young (18-39)** | 14 (31.1) | 14 (56) | | 0 (0) | <0.001 |
|  | **Middle age (40-59)** | 14 (31.1) | 9 (36) | | 5 (25) |  |
|  | **Old age (>60)** | 17 (37.8) | 2 (8) | | 15 (75) |  |
| **Sex** | **Male** | 23 (51.9) | 14 (56) | | 9 (45) | 0.463 |
|  | **Female** | 22 (48.1) | 11 (44) | | 11 (55) |  |
| **Smoking** | **Smoker** | 12 (26.7) | 3 (12) | | 9 (45) | <0.001 |
|  | **Non-smoker** | 33 (73.3) | 22 (88) | | 11 (55) |  |
| **Residence** | **Urban** | 25 (55.6) | 20 (80) | | 5 (20) | <0.001 |
|  | **Rural** | 20 (44.4) | 4 (16) | | 16 (80) |  |
| **Vaccination status** | **Unvaccinated** | 0 (0) | 0 (0) | | 0 (0) | <0.001 |
|  | **Partially vaccinated** | 20 (44.4) | 0 (0) | | 20 (100) |  |
|  | **Full vaccinated** | 14 (31.2) | 14 (56) | | 0 (0) |  |
|  | **+ Booster dose** | 11 (24.4) | 11 (44) | | 1. (0) |  |

-Significant level at P value < 0.05

**Supplementary Table S3: Complete Blood count data of the studied COVID-19 patients**

| **R** | **P-value** | **Severe to critical group**  **N=20** | | **Mild to moderate group**  **N=25** | | | | **The studied laboratory data** | |  |
| --- | --- | --- | --- | --- | --- | --- | --- | --- | --- | --- |
| 0.43** | **<0.000** | 4.5 – 6.5 | | 4.5 – 5.5 | | | | **Range**  **Normal:(**Male: 4.3-5.9 million/mm^3^, Female: 3.5-5.5 million/mm^3^) | | **RBC count** |
|  |  | 5.55±0.74 | | 4.98 ± 0.4 | | | | **Mean ±SD** | |  |
| 0.24* | **<0.000** | 12.5 – 17.5 | | 12.5 – 15.5 | | | | **Range**  **Normal**:(Male: 13.5-17.5 g/dl, Female: 12-16 g/dl) | | **Hb level** |
|  |  | 14.9± 2.2 | | 14.1± 1.1 | | | | **Mean ±SD** | |  |
| -0.627** | **<0.001** | 75000-200000 | | 150000-410000 | | | | **Range** | | **Platelet count**  (cells/μ) |
|  |  | 146000 ± 356600 | | 249800 ± 79700 | | | | **Mean ±SD** | |  |
|  |  | **Thrombocytopenia** | **Normal** | **Normal** | | | | **Type** | **Classification**  **Normal**  (150000-450000/μL)  **Thrombocytopenia**  (<150000/μL) |  |
|  |  | 8 | 12 | 25 | | | | **Count** |  |  |
|  |  | 40% | 60% | 100% | | | | **%** |  |  |
|  |  | 75000 -145000 | 150000-200000 | 150000-410000 | | | | **Range** |  |  |
|  |  | 111250 ±24463 | 169167 **±** 19454 | 249800 ± 79700 | | | | **Mean ±SD** |  |  |
| 0.94* | **<0.000** | 14000 - 20000 | | 3000 -11500 | | | | **Range** | | **Total leukocyte count**  (cells/mm^3^) |
|  |  | 18150 ± 1500.8 | | 7720 ± 2213.96 | | | | **Mean ±SD** | |  |
|  |  | **Leukocytosis** | | **Leukocytosis** | **Leukopenia** | | **Normal** | **Type** | **Classification**  **-Normal TLC**  4-11×10^3^  **-Leukopenia** <4×10^3^  **-Leukocytosis**  >11×10^3^ cells/mm^3^ |  |
|  |  | 20 | | 2 | 2 | | 21 | **Count** |  |  |
|  |  | 100% | | 8% | 8% | | 84% | **%** |  |  |
|  |  | 14000 – 20000 | | 11250-11500 | 3000 - 3500 | | 4500 - 10000 | **Range** |  |  |
|  |  | 18150 ± 1500.8 | | 11325 ± 125 | 3250 ± 250 | | 7761.9± 1868.4 | **Mean ±SD** |  |  |
| 0.09 *** | 0.568 | 350-1440 | | 360-1350 | | | | **Range** | | **Lymphocyte count**  (cells/mm^3^) |
|  |  | 915.2 ± 67.8 | | 867.6 ± 43.5 | | | | **Mean ±SD** | |  |
|  |  | **Lymphopenia** | **Normal** | **Lymphopenia** | | | **Normal** | **Type** | **Classification**  **Normal**  (1200-3500 /mm^3^)  **Lymphopenia**  (<1200/mm^3^) |  |
|  |  | 17 | 3 | 23 | | | 2 | **Count** |  |  |
|  |  | 85% | 15% | 92% | | | 8% | **%** |  |  |
|  |  | 350-1170 | 1350-1440 | 360-1050 | | | 1300-1350 | **Range** |  |  |
|  |  | 833.2 ± 60.9 | 1380 ± 24.5 | 827.8 ± 37.1 | | | 1325 **±** 17.7 | **Mean ±SD** |  |  |
| 0.95 * | **<0.000** | 11900-17000 | | 2625-9200 | | | | **Range** | | **Neutrophil count** |
|  |  | 15333.75±1269 | | 5907 ± 1840 | | | | **Mean ±SD** | |  |
|  |  | **Neutrophilia** | | **Neutrophilia** | | | **Normal** | **Type** | **Classification**  **Normal:**  2500-7500 cells/ μL  **Neutrophilia**  >7500 cells/ μL |  |
|  |  | 20 | | 9 | | | 16 | **Count** |  |  |
|  |  | 100% | | 36% | | | 64% | **%** |  |  |
|  |  | 11900-17000 | | 7200-9200 | | | 2625-7000 | **Range** |  |  |
|  |  | 15333.75±1269 | | 7786.1 ± 575.8 | | | 4850 ± 1414.7 | **Mean ±SD** |  |  |
| 0.95 * | **<0.000** | 280-800 | | 70-400 | | | | **Range** | | **Monocyte count** |
|  |  | 726 ± 60 | | 224 ± 89.3 | | | | **Mean ±SD** | |  |
|  |  | **Normal** | | **Monocytopenia** | | | **Normal** | **Type** | **Classification**  **Normal (**200-800 cells/ μL)  **Monocytopenia (**<200 cells/ μL) |  |
|  |  | 20 | | 13 | | | 12 | **Count** |  |  |
|  |  | 100% | | 52% | | | 48% | **%** |  |  |
|  |  | 280-800 | | 275-400 | | | 70-190 | **Range** |  |  |
|  |  | 726 ± 60 | | 301.5 ± 59.5 | | | 152.7 ±38 | **Mean ±SD** |  |  |
| 0.73 | **<0.001** | 10.6 – 41.3 | | 2.8 -13 | | | | **Range** | | **NLR** |
|  |  | 19.2 **±** 8 | | 7.8 ± 2.4 | | | | **Mean ±SD** | |  |
|  |  | **Elevated** | | **Elevated** | | **Normal** | | **Type** | **Classification**  **Normal**  (1-3)  **Elevated**  (>3) |  |
|  |  | 20 | | 23 | | 2 | | **Count** |  |  |
|  |  | 100% | | 92% | | 8% | | **%** |  |  |
|  |  | 10.6 – 41.3 | | 4 -13 | | 2.8 -3 | | **Range** |  |  |
|  |  | 5.8 **±** 6.5 | | 7.5 **±** 2.5 | | 2.9 **±**0.1 | | **Mean ±SD** |  |  |

**Supplementary Table S4: Comparison of D-dimer and CRP level among different degrees of disease severity.**

| **R** | **P-value** | **Sever to critical**  **N=20** | **Mild to moderate**  **N=25** | | **The studied laboratory data** | | |
| --- | --- | --- | --- | --- | --- | --- | --- |
| 0.519* | <0.001 | 0.8 -32 | 0.2 -1.2 | | **Range** | | **D-dimer serum level** |
|  |  | 5.8 **±** 6.5 | 0.5 ± 0.29 | | **Mean ±SD** | |  |
|  |  | **Elevated** | **Elevated** | **Normal** | **Type** | **Classification**  **Normal**  (0-0.5mg/ml)  **Elevated**  (>0.5mg/ml) |  |
|  |  | 20 (100) | 8 (32) | 17 (68) | **N (%)** |  |  |
|  |  | 0.8 -32 | 0.6-1.2 | 0.2-0.5 | **Range** |  |  |
|  |  | 5.8 **±** 6.5 | 0. 9 **±** 0.2 | 0.37**±**0.1 | **Mean ±SD** |  |  |
| 0.880* | <0.001 | 27.7-76.8 | 2.6-22.3 | | **Range** | | **CRP serum level** |
|  |  | 49.6±13.6 | 11±6.7 | | **Mean ±SD** | |  |
|  |  | **Elevated** | **Elevated** | | **Type** | **Classification**  **Normal**  (0.3-1 mg/dl)  **Elevated**  (>1 mg/dl) |  |
|  |  | 20 (100) | 25 (100) | | **N (%)** |  |  |
|  |  | 27.7-76.8 | 2.6-22.3 | | **Range** |  |  |
|  |  | 49.6±13.6 | 11±6.7 | | **Mean ±SD** |  |  |

Significant level at P value < 0.05

* r (0.7-1) = strong correlation

**r (0.3-0.7) = moderate correlation


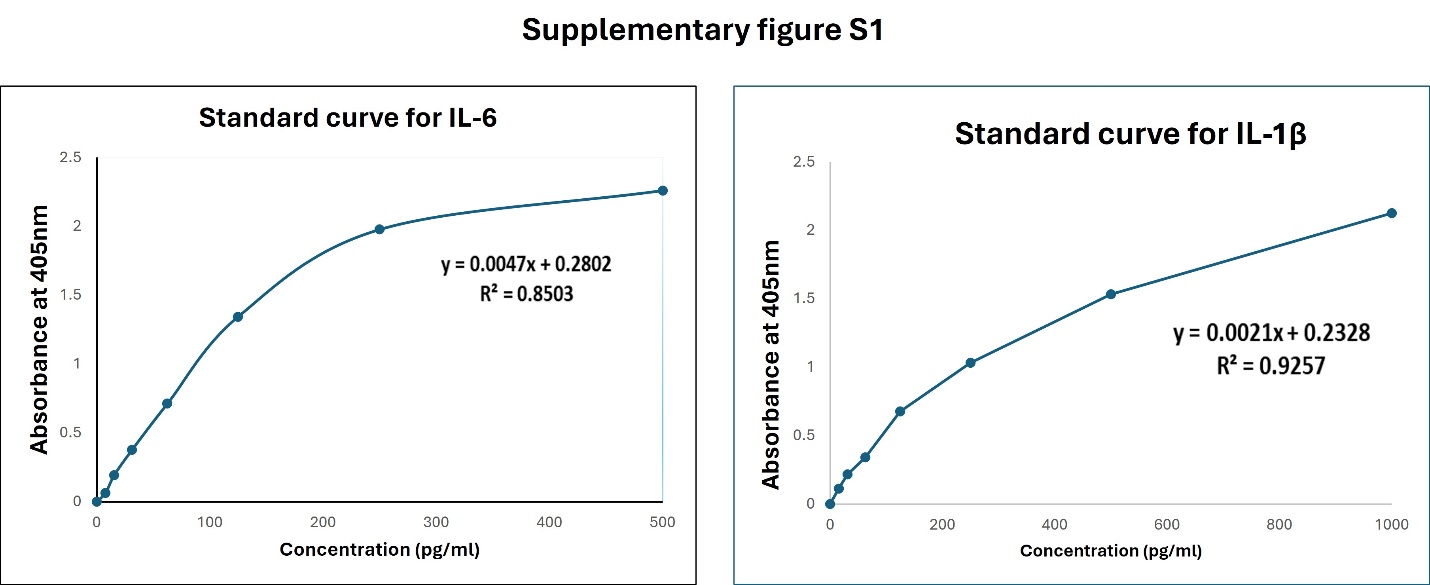

Supplement: Supplementary file 1 — Supplementary Information. [file 41598_2025_34444_MOESM1_ESM.docx]
